# Urticaria in a Pediatric Population: A Portuguese Single-Center Cohort Report

**DOI:** 10.7759/cureus.26659

**Published:** 2022-07-08

**Authors:** Inês Coelho, Bárbara Neto, Diana Bordalo, Sylvia Jacob

**Affiliations:** 1 Pediatrics, Centro Hospitalar de São João, Porto, PRT; 2 Pediatrics, Centro Hospitalar Universitário do Algarve, Faro, PRT

**Keywords:** skin conditions, pediatric, retrospective cohort, chronic spontaneous urticaria, urticaria

## Abstract

Background

Urticaria typically involves the skin and mucosa and is characterized by the development of wheals, angioedema, or both. According to the temporal evolution of the lesions, urticaria is classified as acute (AU) or chronic (CU), depending on whether the episodes last for fewer or more than six weeks, respectively. This study aimed to characterize a group of children and adolescents with urticaria and describe its subtypes, associated comorbidities, treatment, and evolution.

Methodology

This retrospective, observational study included patients aged <18 years who were diagnosed with urticaria in a tertiary teaching hospital in Portugal, and followed up in a Pediatric Allergy Unit, between January 2019 and December 2021.

Results

A total of 43 patients, aged nine months to 16 years were included. Of these, 22 (51%) were males. AU was identified in 12 (28%) cases, chronic spontaneous urticaria in 21 (63%), and physical urticaria (to cold) in four (9%). Autoantibodies were detected in four patients with spontaneous urticaria. In 6% of patients with CU, the episodes were associated with angioedema. Most CU episodes were successfully managed with the recommended or double the recommended dose (48%) of H1 antihistamines. Three patients requiring fourfold higher than the recommended dose of H1 antihistamines remained unresponsive and were started on omalizumab. Associated autoimmune thyroiditis was diagnosed in four patients.

Conclusions

In this cohort of patients, urticaria was equally distributed between the genders and the first-line therapy was second-generation antihistamines, consistent with current guidelines. Universal screening for autoimmune diseases in patients with chronic spontaneous urticaria revealed four cases of thyroiditis, which supports the relevance of this approach when managing CU.

## Introduction

Urticaria typically involves the skin and mucosa and is characterized by the development of wheals, angioedema, or both. The lesions transiently disappear or attenuate to digital pressure and regress, spontaneously or with therapy, with no residual lesion, within less than 24 hours [1-4]. In approximately 50% of the cases, it is accompanied by angioedema, which reflects the involvement of the deeper structures of the dermis [1-4]. According to the temporal evolution of the lesions, urticaria is classified as acute (AU) or chronic (CU), depending on whether episodes last for fewer or more than six weeks, respectively [1,4-6].

The incidence of all forms of urticaria in children varies between 2.1% and 6.7%, being more frequent in younger children [2,5]. CU in children/adolescents is uncommon, occurring in only 0.1-0.3%, a lower percentage than that observed in adults [7,8]. Although much of the knowledge about adult urticaria may be applied to children and adolescents, the uniqueness inherent to this age group, especially with regard to etiology and evolution, is still poorly studied [1,6-9]. This study aimed to characterize a group of children and adolescents with urticaria and describe its subtypes, associated comorbidities, treatment, and evolution.

## Materials and methods

This retrospective, observational study included patients aged <18 years who were diagnosed with urticaria in a tertiary teaching hospital in Portugal and followed up in a Pediatric Allergy Unit. The study was performed between January 2019 and December 2021. Children with episodes of AU or CU, with or without an identified etiology (spontaneous, physical, or other specific types), were included, and children with episodes of urticaria associated with food allergy were excluded. The new classification of urticaria was considered, according to the recommendations proposed by the Dermatology Section European Academy of Allergy and Clinical Immunology (EAACI), Global Allergy and Asthma European Network (GA2LEN), European Dermatology Forum (EDF), and World Allergy Organization (WAO) in 2018 [1].

Given that these retrospective data are presented as a deidentified case series, we did not request Institutional Review Board approval. All patients had a general consent signed by a parent/legal guardian permitting their information in the medical record to be used for research. Data regarding anamnesis and physical examination were analyzed, as well as the results of the complementary diagnostic tests, which could include skin tests on food and common inhalants; specific tests for confirmation of physical urticaria in suspected cases (application of pressure on the skin, test with ice cube, contact with water or physical exercise); blood tests; total and specific immunoglobulin (Ig)E (according to clinical suspicion); thyroid function and antibodies; antinuclear antibodies (ANA) and complement study (CH50, C3, C4, and C1 inhibitor in case of associated angioedema); serologies (Epstein-Barr virus (EBV), cytomegalovirus (CMV), herpes simplex (HSV), parvovirus, *Helicobacter pylori*); urine examination and culture; and stool parasitological examination.

The demographic and clinical variables analyzed included sex, age, clinical manifestations, identified triggering factors, comorbidities, complementary diagnostic tests, treatment, and evolution. The data obtained were analyzed using the Sigmastat 3.5 (Systat Software, San Jose, CA, USA) statistical analysis using descriptive analysis.

## Results

Demographic data

Of the 43 children, 22 (51.2%) were males. The median age at the time of symptom onset was six years (ranging from nine months to 16 years).

Classification

AU was identified in 12 (27.9%) cases and CU in 31 (72.1%) cases. In patients with CU, 28 (87.1%) cases corresponded to spontaneous urticaria and four (12.9%) to cold-induced urticaria (Table 1). In two (6.5%) patients, the episodes were associated with angioedema.

**Table 1:** Subtypes of urticaria identified in the studied sample.

Classification		Absolute number (43)	Percentage
Acute urticaria		12	27.9%
Chronic urticaria	Spontaneous	27	87.1%
	Cold-induced	4	12.9%

Clinical manifestations, history, and physical examination

Of the 12 patients with AU, eight (66.7%) had a previous viral infection, and in the remaining four (33.3%) cases, no associated triggers were found. At the time of the appointment, on physical examination, none had any remaining lesions.

Of the 31 patients with CU, the frequency of symptoms was well characterized, appearing daily in three (9.7%) and once a week in five (16.1%) (Table 2). In the remaining 23 (74.2%) cases, there were references to more sporadic flares. In total, 21 (67.7%) children/adolescents had asthma and/or associated rhinitis, with evidence of sensitization to aeroallergens in 13 cases. Thirteen (41.9%) patients had atopic dermatitis. Two (6.5%) patients had a family history of CU, although some with different subtypes. On physical examination, dermographism was observed in 10 (32.3%) patients.

**Table 2:** Frequency, history, and physical examination of chronic urticaria.

Chronic urticaria		
Frequency	Daily	3 (9.7%)
	Once a week	5 (16.1%)
	Sporadic flares	23 (74.2%)
Personal history	Asthma and/or rhinitis	21 (67.7%)
	Atopic dermatitis	13 (41.9%)
Family history	Chronic urticaria	2 (6.5%)
Physical examination	Dermographism	10 (32.3%)

Complementary diagnostic tests

In terms of etiology in AU, eight patients had serological evidence of infection by group A *Streptococcus*, CMV, EBV, and/or HSV.

Regarding CU, three patients had positive IgG for *Helicobacter pylori *with no reference to digestive symptoms, hence, upper gastrointestinal endoscopy was not performed for these patients. In two children, the parasitological examination of stool was positive for *Entamoeba coli*. In the investigation carried out to exclude autoimmune pathology, positive tissue transglutaminase IgA was identified in one patient. In four children/adolescents with spontaneous urticaria, thyroid antibodies were detected, all with normal thyroid function at the beginning of the investigation, but on follow up they showed hypothyroidism (Table 3). No quantitative or functional complement change was detected in patients with angioedema. In four children/adolescents with suspected cold-induced urticaria, the provocation test with an ice cube was carried out, which was positive in all cases.

**Table 3:** Results of complementary diagnostic tests in chronic urticaria. Ig: immunoglobulin

Complementary diagnostic tests in chronic urticaria	
IgG *Helicobacter pylori*	3 (9.7%)
*Entamoeba coli* in parasitological examination of stool	2 (6.5%)
Tissue transglutaminase IgA	1 (3.2%)
Thyroid antibodies	4 (12.9%)

Treatment

All patients were treated with second-generation antihistamines as first-line therapy. The patients with AU were asymptomatic after treatment. In the CU group, most episodes were successfully managed with the recommended or double the recommended dose (48%) of H1-antihistamines. Three patients requiring fourfold higher than the recommended dose of H1-antihistamines remained unresponsive and were started on omalizumab.

Evolution

Of all 12 patients with AU, there was remission of urticaria in the first month after the onset of symptoms. Of 24 (77.4%) patients with CU whose evolution was known, there was remission of episodes of urticaria during the first three years of the disease. Remission of symptoms appeared on average 12 months after the onset of symptoms.

## Discussion

In this cohort of children and adolescents with urticaria, there was an equal distribution between genders, contrary to what happens in adults, where there is a predominance of females [2,5,10,11]. In this cohort, there was a low percentage of cases with associated angioedema (6%), divergent from what is described in the literature. Previous studies refer to the occurrence of urticaria isolated in 48.8-78.4% of cases and associated urticaria angioedema in 15.0-50.4% [5,11,12]. In a quarter of the cases (25.8%), the symptoms appeared on a daily or weekly basis; however, published series reveal values greater than 50% [11].

Most cases corresponded to spontaneous CU previously called chronic idiopathic urticaria. This type of CU is the most frequent in pediatric patients [3]. Among physical urticaria subtypes, cold urticaria was the only one described in this cohort. Cold urticaria, although very rare in adults, is relatively frequent in children and adolescents, as was observed in the analyzed sample, corresponding to 12.9% of the CU cases. Anamnesis was fundamental in identifying triggering factors in four cases and guided the diagnosis regarding the type of urticaria. This leads to the provocation test with the ice cube, which confirmed the diagnosis of cold urticaria.

Approaching patients with spontaneous urticaria was difficult, given that no triggering factors were identified on anamnesis. In this regard, it is important to keep in mind that autoimmune diseases, such as autoimmune thyroiditis, celiac disease, type 1 diabetes mellitus, inflammatory bowel disease, juvenile idiopathic arthritis, and systemic lupus erythematosus can appear before, at the same time, or after the appearance of urticaria episodes [3,5,13,14]. Among the 27 cases of spontaneous urticaria, autoimmune phenomena were identified in five (18.5%): one with positive tissue transglutaminase IgA (3.7%) and four with positive thyroid antibodies (14.8%), although all without other symptoms and with normal thyroid function.

In a Canadian study carried out with 624 CU patients, autoimmune thyroiditis was diagnosed in 90 (about 14%) patients, a higher value than that found in the control group (3-6%) and similar to that found in the presented sample [14]. Infectious diseases that have been associated with CU and that seem to be triggering/aggravating factors of episodes of urticaria include *Escherichia coli* urinary tract infection, upper respiratory tract, *Helicobacter pylori*, CMV, EBV, and parasitic infections [5,15-17].

In adults, the implication of *Helicobacter pylori* in the etiology of CU has been reported, although with much controversy [18-22]. Despite the positive serology for IgG in three patients, none had digestive symptoms, so they had no indication to perform upper gastrointestinal endoscopy. Even asymptomatic, all three were treated for *Helicobacter pylori* but the treatment had no impact on urticaria and they maintained the frequency and intensity of the urticaria flares. The genetic factors appear to play some role in the etiology of CU, given the evidence of positive family history in 6.5% of the cohort, similar to other studies (10.0%) [15].

The approach to patients with CU is mainly based on a complete anamnesis and physical examination to characterize the clinical manifestations and identify possible triggering factors [4,23]. The latest recommendations from the EAACI, GA2LEN, EDF, and WAO consider that there is no need for additional routine investigation, beyond the provocation tests that can be performed in some cases if the clinical history data point to the diagnosis of urticaria triggered by a well-identified factor [1,6,23]. The exception should be made in cases of cold-induced urticaria, in which complete blood count and sedimentation rate/C-reactive protein should be performed. In cases of spontaneous CU, a more extensive complementary study should be carried out to screen for some diseases (autoimmune, infectious) that may be associated. This group of complementary examinations may include complete blood count, sedimentation rate, lactic dehydrogenase, liver enzymes, renal function, urine test and urine culture, parasitological examination of stool, ANA, thyroid antibodies and function, screening for celiac disease, and skin tests if food allergy is suspected [1,3]. Most of the time, the results of these tests are normal, as observed in 17 (62.9%) of the 27 cases of spontaneous CU identified in the studied sample, a similar percentage to that shown in a study with 130 patients, in which 68% had normal results [24]. The main measure to be taken in cases of urticaria with an identified trigger is to avoid exposure to the trigger [3,4].

Second-generation antihistamines (cetirizine, desloratadine, levocetirizine) constitute the first-line treatment in all types of urticaria [3]. In refractory cases, up-dosing the initially proposed antihistamine up to fourfold is recommended. If inadequate control persists, another drug must be associated. In such settings, the second-line therapy should be omalizumab (anti-IgE) and the third-line ciclosporin A [1]. Omalizumab is effective and safe in the treatment of CU. Although ciclosporin A has a moderate, direct effect on mast cell mediator release, it is off-label and is recommended only for patients with severe disease refractory to any dose of antihistamine and omalizumab in combination [1]. Oral corticosteroids can shorten the duration of episodes, but they are not a good therapeutic option due to the associated side effects and the fact that they can mask other etiologies/diseases [3].

In this study, most episodes were successfully managed with the recommended or double the recommended dose (48%) of H1-antihistamines. Three patients with CU requiring fourfold higher than the recommended dose of H1 antihistamines remained unresponsive and were started on omalizumab, which illustrates the difficulty in controlling the disease in some cases.

Regarding the evolution of CU, in 12 patients there was remission of symptoms, on average 12 months after diagnosis. Khakoo et al. studied 53 children with physical urticaria and showed that in only 11.6% of the cases the disease had resolved after one year and in 38.4% after five years, which shows that physical urticaria may have a worse prognosis [25]. The authors also showed that a history of allergy and more frequent episodes (daily/weekly) were related to a worse prognosis (lower percentage of remission and later remission) [25]. The number of patients with physical urticaria in the presented sample was not sufficient to assess these variables.

The authors acknowledge the limitations of this study, particularly the single-center, retrospective design, and the small number of patients collected from a specialty practice. This study aims to contribute to our understanding of urticaria describing the experience at one tertiary care health center. Further research is needed to clarify the management and prognosis in pediatric patients.

## Conclusions

The obtained data emphasize that a complete anamnesis and physical examination are essential in identifying possible triggering factors and in classifying urticaria subtypes. In this cohort of patients, urticaria was equally distributed between genders and the first-line therapy was second-generation anti-histamines, consistent with current guidelines.

Universal screening for autoimmune diseases in patients with chronic spontaneous urticaria revealed four cases of thyroiditis, which supports the relevance of this approach when managing CU. Although urticaria may be associated with or triggered by infectious diseases, this does not alter the therapeutic approach. In addition, besides not seeming to play a relevant role in the etiology of CU, especially in children, *Helicobacter pylori* infection should be investigated only in the presence of symptoms suggestive of that diagnosis. In 12 patients, spontaneous resolution of symptoms occurred on average up to 12 months after the diagnosis. Despite this, it was necessary to maintain daily second-generation antihistamines to control symptoms in a high number of cases.
